# Role of Sense of Coherence in Mediating the Relationship Between End-of-Life Caregiving Burden and Bereavement Depressive-Symptom Trajectory

**DOI:** 10.1097/jnr.0000000000000731

**Published:** 2026-03-11

**Authors:** Fur-Hsing Wen, Wen-Chi Chou, Ming-Mo Hou, Po-Jung Su, Wen-Chi Shen, Jen-Shi Chen, Wen-Cheng Chang, Siew Tzuh Tang

**Affiliations:** 1 Department of International Business, Soochow University, Taipei, Taiwan, ROC; 2 Division of Hematology-Oncology, Chang Gung Memorial Hospital at Linkou, Taoyuan City, Taiwan, ROC; 3 College of Medicine, Chang Gung University, Taoyuan, Taiwan, ROC; 4 School of Nursing, Chang Gung University, Taoyuan, Taiwan, ROC; 5 Department of Nursing, Chang Gung Memorial Hospital at Kaohsiung, Taiwan, ROC; 6 Department of Nursing, Chang Gung University of Science and Technology, Taoyuan, Taiwan, ROC

**Keywords:** sense of coherence, mediation, depressive symptoms, caregiving burden, bereavement

## Abstract

**Background:**

The results of meta-analyses indicate sense of coherence (SOC) has a positive effect in terms of reducing subjective caregiving burden and depressive symptoms in family caregivers. However, the mechanism by which SOC mediates psychological distress remains unexplored longitudinally.

**Purpose:**

This study was designed to examine how SOC mediates the impact of self-perceived caregiving burden on depressive symptoms during the first 2 years of bereavement following the care-recipient’s death.

**Methods:**

For this secondary analysis, 649 individuals who had provided end-of-life caregiving to cancer patients were recruited and enrolled as participants. Their appraisal of caregiving burden over the patient’s last 6 months was categorized into four (slight, mild, moderate, and severe) trajectories based on the Caregiver Reaction Assessment (CRA) score. Using multinomial logistic regression, how SOC mediates the relationship between CRA trajectories and seven depressive-symptom trajectories (minimal-impact-resilience, recovery, preloss-only, delayed-symptomatic, relief, prolonged-symptomatic, chronically persistent distressed) was examined using the three recommended steps for mediation analysis.

**Results:**

The largest percentage (27.6%) of participants endured moderate-to-severely high end-of-life (EOL) caregiving burden, and one-fourth (24.0%) experienced distressing depressive-symptom trajectories (i.e., delayed symptomatic, relief, prolonged symptomatic, and chronically persistent distressed) from EOL caregiving over the first two bereavement years. Unlike the more severe depressive-symptom trajectories, the minimal-impact-resilience and recovery trajectories were shown to be associated with having a stronger SOC. Furthermore, SOC mediation was found to eliminate or reduce the risks for the most distressing depressive-symptom trajectories (preloss-only, delayed-symptomatic, relief, prolonged-symptomatic, and chronically persistent-distressed), whereas the effects on milder trajectories (minimal-impact-resilience and recovery trajectories) were weaker. The SOC-mediating effect was more pronounced among those participants in the severe burden CRA trajectory than their peers in the slight-to-moderate caregiving burden trajectories.

**Conclusions/Implications for Practice:**

Based on the findings, SOC completely or partially mediates the effects of end-of-life caregiving burden on caregiver depressive symptoms during the first two bereavement years following care-recipient death. Strengthening SOC—especially in heavily burdened caregivers—through high-quality end-of-life care may ease caregiving burden and reduce the psychological distress of caregiving during the bereavement period.

## Introduction

Family caregivers play a vital role in providing end-of-life care to cancer patients (Coumoundouros et al., 2019; Ornstein et al., 2017), helping offset the substantial societal costs of formal care services (Coumoundouros et al., 2019; Higginson, et al., 2020; Ornstein et al., 2017). Despite this contribution, caregivers face significant and competing end-of-life caregiving demands that lead to considerable physical (Hopps et al., 2017; Uccheddu et al., 2019), psychological (e.g., depressive symptoms; Trevino et al., 2018; Uccheddu et al., 2019), and financial (Hopps et al., 2017; Iragorri et al., 2021) strains. These burdens frequently persist into bereavement (Große et al., 2018; Blanner Kristiansen et al., 2019), contributing to elevated risks of depressive disorders that may negatively impact caregiver health (Blanner Kristiansen et al., 2019; Rajan et al., 2020), social relationships, workplace relationships (Rajan et al., 2020), and the broader health care system (Rajan et al., 2020; König et al., 2019).

The findings of decades of research indicate that not all bereaved caregivers suffer from the detrimental consequences of end-of-life caregiving and bereavement and that, while most overcome these highly stressful challenges, a minority adjust maladaptively (Aneshensel et al., 2004; Bonanno et al., 2002; Bonanno et al., 2015; Galatzer-Levy & Bonanno, 2012; Galatzer-Levy et al., 2018; Szabó et al., 2020). Research findings suggest personal strength/resilience is an important factor (Aneshensel et al., 2004; Bonanno et al., 2002; Bonanno et al., 2015; Galatzer-Levy & Bonanno, 2012; Galatzer-Levy et al., 2018; Szabó et al., 2020). Resilience refers to the capacity to adapt to or bounce back from stress/adversity through the activation of internal coping resources, enabling stable and healthy psychological functioning and positive outcomes despite challenging circumstances (Bonanno et al., 2015; Kalisch et al., 2017). Among multiple related but distinct personal-strength/resilience or health-benefitting concepts, Antonovsky’s sense of coherence (SOC; Antonovsky, 1993) is notable.

SOC, comprising the three elements of comprehensibility, manageability, and meaningfulness (Antonovsky, 1993), is a global orientation reflecting sustained confidence that (a) stimuli from internal and external environments are structured, predictable, and understandable (comprehensibility); (b) sufficient resources are available to meet the demands of these stimuli (manageability); and (c) these demands are meaningful challenges worth engaging with (meaningfulness). SOC provides a framework for understanding the coping processes that foster resilience, helping individuals appraise stressful events as more comprehensible, manageable, and meaningful (Antonovsky, 1993). Moreover, SOC has further been suggested to protect individuals from the negative effects of life’s adversities over other resistance factors (Antonovsky, 1993), e.g., personal-strength/resilience or health-benefitting concepts (Hochwälder, 2019; Schäfer, 2020). SOC has consistently shown incremental validity over trait-resilience, optimism, and self-compassion in the contexts of psychological distress (Grevenstein et al., 2016) and physical-health-related quality of life (Trapp et al., 2015). Also, SOC provides value beyond trait-resilience, hardiness, locus of control, sense of mastery, self-efficacy, and dispositional optimism in posttraumatic stress disorder (Schäfer, 2020).

Specifically, SOC has been shown in meta-analysis studies to moderately negatively impact subjective caregiving burden and moderately to significantly reduce symptoms of depression and anxiety across several caregiving populations (Del-Pino-Casado et al., 2019), as well as to strongly protect against burnout in caregivers (Gérain & Zech, 2019). Although resilience and coping are dynamic processes (Kalisch et al., 2017), the focus of most studies conducted to examine the association between SOC and caregiving outcomes has been cross-sectional (Del-Pino-Casado et al., 2019), limiting insight into the evolving nature of coping with end-of-life caregiving and the potential or actual loss of a loved one. Moreover, cross-sectional studies are inherently unable to elucidate the temporal influence of SOC (Schäfer et al., 2019) on caregiving and bereavement outcomes. This limitation may threaten the validity of meta-synthesized findings (Del-Pino-Casado et al., 2019; Gérain & Zech, 2019) by introducing the risk of reverse causation (Mittelmark et al., 2022; Schäfer et al., 2019). Moreover, most studies, including the referenced meta-analysis (Del-Pino-Casado et al., 2019), have focused solely on SOC’s main effect on caregiving outcomes. While these effects appear robust despite confounders (Del-Pino-Casado et al., 2019), further research, particularly longitudinal studies (Mc Gee et al., 2018), is needed to explore the inadequately studied issue of how SOC influences or mediates the impact of caregiving burden on psychological distress in caregivers (Mc Gee et al., 2018; Mittelmark et al., 2022). Evidence from a meta-analysis (Del-Pino-Casado et al., 2019) of primarily cross-sectional studies indicates that SOC has a moderately negative association with subjective caregiving burden and a moderate to strongly negative association with depressive symptoms. This suggests that the stress from end-of-life caregiving and the impending or actual loss of a loved one may weaken SOC in caregivers and potentially lead to more intense or prolonged depressive symptoms. Longitudinal studies conducted to explore how SOC mediates the link between self-perceived end-of-life caregiving burden and bereavement-related psychological distress (e.g., depressive symptoms) may clarify the potential of SOC as a target for interventions (Guo et al., 2024; Langeland et al., 2022; Mittelmark et al., 2022) that may help facilitate caregiver adjustment to the intertwined stressors of end-of-life caregiving and bereavement and potentially reduce the risk of profound and prolonged depressive symptoms.

A recent scoping review of 41 studies (Langeland et al., 2022) demonstrated the effectiveness of various interventions in enhancing SOC across diverse populations, including individuals with different diagnoses and physical impairments, despite SOC being theorized as a relatively stable trait (Antonovsky, 1993). Most studies included in a recent 18-study systematic review found salutogenic-based interventions for older adults have an enhancement effect on SOC, quality of life, self-efficacy, self-management, sense of life meaning, and mental health (Guo et al., 2024).

Therefore, this secondary analysis was developed to examine whether SOC mediates the effect of self-perceived end-of-life (EOL) caregiving burden on caregiver risk of following more distressing depressive-symptom trajectories during the first 2 years following care-recipient death. If a significant mediating effect is found, SOC may offer a key target for future interventions to mitigate psychological distress.

## Methods

### Research Design and Sample

Details of this secondary-analysis study, which used data from two longitudinal studies, i.e., one examining associations between end-of-life care and depressive symptoms in caregivers (Kuo et al., 2019) and the other evaluating the effects of an advanced care planning intervention on these symptoms (Tang et al., 2019), have been reported previously (Wen et al., 2021; Wen et al., 2022), i.e., the study design, purpose, participant eligibility, subject enrollment, participation in pre- and postloss surveys, measures, and identification (Wen et al., 2021) and predictors (Wen et al., 2022) of distinct depressive-symptom trajectories from end-of-life caregiving through the first 2 years of bereavement. Six hundred sixty-one caregivers of cancer patients receiving EOL care at a medical center in northwest Taiwan were recruited from 2010 to 2017 and were followed through their first two bereavement years (until July 2020). Only 649 participants were included in the final analysis due to incomplete data collected from 12 of the participants. Most of the participants were female (71.1%) and either the care-recipient’s spouse or adult child (85.5%). Their mean age was 52.14 (*SD* = 2.67) years. The participants completed in-person interviews on caregiving experiences and SOC at enrollment and were followed up with on a monthly basis until the end of data collection. Depressive symptoms were assessed postloss via phone or mail at 1, 3, 6, 13, 18, and 24 months.

### Ethical Consideration

This study received approval from the central institutional review board of Chang Gung Medical Foundation (No: 98-0476B, 101-0898A3). Each family surrogate signed informed consent before participation.

### Measures

#### Outcome Variables

Depressive symptoms were assessed using the 20-item Center for Epidemiologic Studies Depression Scale (CES-D; Radloff, 1977), which includes four subscales: positive emotions, depressive emotions, physical activity, and social difficulties. Total possible scores for the CES-D range from 0 to 60, with higher scores indicating greater symptom severity (Radloff, 1977) and scores above 16 indicating severe depressive symptoms (Radloff, 1977).

Using latent class growth analysis, seven depressive-symptom trajectories based on symptom timing, intensity, and duration were identified across the EOL-caregiving-through-2-years-of-post-death-bereavement period. These trajectories and their respective prevalences are minimal-impact resilience (20.4%), recovery (34.0%), preloss-only (21.6%), delayed symptomatic (9.1%), relief (5.9%), prolonged symptomatic (6.5%), and chronically persistent distressed (2.5%; Wen et al., 2021). The minimal-impact resilience group shows consistently low symptoms with minor fluctuations around the patient’s death. In the recovery group, symptoms exceeded the CES-D threshold preloss, peaking 1 month postloss and then declining below threshold by 6–7 months. The preloss-only group shows mild to moderate symptoms during caregiving that quickly subsides after loss. The delayed symptomatic group begins with mild symptoms that peak around death, declining postloss and rising again after 18 months. In the relief group, symptoms intensify during caregiving but drop significantly near death and fall below the threshold by 6–7 months. The prolonged symptomatic group experiences moderate to severe symptoms before loss, peaking postloss, and then steadily declining and resolving. The chronically persistent distressed group shows severe symptoms throughout, with some improvement postloss, although levels remain well above threshold. The complete figure illustrating these trajectories has been published (Wen et al., 2021).

#### Independent Variable

Modifiable factors predisposing caregivers to distinct depressive-symptom trajectories (Wen et al., 2022) were identified in three domains based on the stress–appraisal–coping model (Lazarus & Folkman, 1984). These included (a) stressors (objective caregiving demands), (b) stress appraisal (appraisal of EOL-caregiving burden), and (c) available resources (internal coping capacity and external social support). Building on the current authors’ previous finding of SOC’s main effect (Wen et al., 2022), SOC was examined in this study as a potential mechanism linking stress appraisal of end-of-life caregiving burden to being included in the seven identified depressive-symptom trajectories (Wen et al., 2021). Participants’ appraisal of end-of-life caregiving burden was assessed using the 24-item Caregiver Reaction Assessment (CRA; Given et al., 1992), which is widely used to evaluate the impact of this burden on caregivers’ schedules, health, finances, self-esteem (positive or resentful), and perceived lack of family support. Total possible scores for the CRA range from 24 to 120, with higher scores indicating more negative appraisals of caregiving impact.

Participant responses to the CRA varied over time during end-of-life caregiving, making this a time-varying variable. To account for this, latent class growth analysis was used with total CRA scores as a continuous latent class indicator to identify CRA trajectories over the patient’s last 6 months of life (Wen et al., 2022). Model fit, parsimony, sample size, and clinical interpretability guided the selection of a four-trajectory solution, with each trajectory representing a homogenous group of caregivers with similar CRA score levels, labeled (prevalence) as slight (15.4%), mild (57.0%), moderate (20.5%), and severe (7.1%). Details on the model selection process are reported elsewhere (Wen et al., 2022).

#### Mediator

The coping capacity of the participants was measured using the 13-item SOC scale (Antonovsky, 1993) at the first assessment, which was conducted during the patient’s last 6 months of life. Total possible SOC scale scores range from 13 to 91, with higher scores indicating stronger SOC.

### Statistical Analysis

To test whether SOC mediated the associations between CRA trajectories and depressive-symptom trajectories from end-of-life caregiving through the two bereavement years, a three-step approach for mediation analysis (Baron & Kenny, 1986) was applied using multinomial logistic regression, which is appropriate for categorical outcome variables with more than two levels. First, the association between CRA trajectories (independent variable) and preloss SOC scores (mediator) was assessed using general linear modeling to determine whether different CRA trajectory groups exhibited significantly different SOC levels. Second, the significance of the association between CRA trajectories and depressive-symptom trajectories (outcome) was assessed. In the third step, SOC was included as a covariate in the multinomial logistic regression model to evaluate whether its inclusion attenuated the associations between CRA trajectories and depressive-symptom trajectories. Complete mediation was indicated if the CRA–depressive symptom relationship became statistically nonsignificant after adjusting for SOC. Partial mediation was suggested if the strength of the association (i.e., the odds ratios) between CRA and depressive-symptom trajectories increased and statistical significance (*p* values) increased after controlling for SOC. Regression estimates were exponentiated to yield a*OR*s with 95% confidence intervals (CIs).

## Results

### Association Between EOL Caregiving Burden Trajectories and SOC

CRA trajectories were found to be significantly associated with SOC. The participants in less-burdened CRA trajectories, i.e., lower negative impact from caregiving, reported significantly higher levels of SOC than those in the severe CRA trajectory (Table 1).

**Table 1:** Association Between End-of-Life Caregiving Burden Trajectories and Sense of Coherence at the Initial (EOL Care Period) Assessment (*N* = 649)

CRA Trajectory	Trajectory Size	CRA Score		SOC Score		β	95% CI	*p*
	*n* (%)	Mean	*SD*	Mean	*SD*			
Slight	100 (15.4)	52.04	6.70	74.01	13.61	34.24	[29.03, 39.45]	<.001
Mild	370 (57.0)	62.99	5.90	63.45	14.41	23.83	[19.26, 28.41]	<.001
Moderate	133 (20.5)	73.37	6.68	50.67	15.25	10.93	[5.98, 15.88]	<.001
Severe	46 (7.1)	86.87	8.42	39.65	15.44	reference		

*Note.* Appraisal of participant end-of-life caregiving burden was measured using the Caregiver Reaction Assessment (CRA). Sense of coherence (SOC) was measured using the SOC scale.

Multiple regression analysis adjusted for gender and age. Outcome variable: SOC score; independent variable: CRA trajectory. CI = confidence interval.

### Associations Between EOL Caregiving Burden and Depressive-Symptom Trajectories From EOL Caregiving Through the First 2 Bereavement Years

Multinomial logistic regressions showed a significant association between CRA trajectories and inclusion in depressive-symptom trajectories (see top panels of Tables 2–5). Of the 57 comparisons, 31 were statistically significant. Adjusted odds ratios (a*OR*s) compared the likelihood of inclusion in the depressive-symptom trajectory based on CRA trajectories, with the severe CRA trajectory (Trajectory 4) used as the reference group. Compared with those in the reference group, the participants in the slight and mild CRA trajectories were less likely to be in the relief, prolonged-symptomatic, and chronically persistent-distressed trajectories than the minimal-impact resilience, recovery, preloss-only, and delayed-symptomatic trajectories (Tables 2–5). In addition, those in the slight and mild trajectories were less likely to be in the preloss-only trajectory than the minimal-impact resilience and recovery trajectories (Tables 2 and 3). Also, the participants in the slight CRA trajectory were less likely to be in the delayed-symptomatic trajectory than the minimal-impact resilience trajectory (Table 2). Finally, compared with their peers in the severe CRA trajectory, the participants in the moderate CRA trajectory were less likely to be in the relief, prolonged-symptomatic, and chronically persistent-distressed trajectories than the recovery and preloss-only trajectories (Tables 3 and 4), and less likely to be in the relief trajectory than the minimal-impact resilience trajectory (Table 2).

**Table 2:** Mediation Effects of Preloss SOC on Associations Between Self-Perceived End-of-Life Caregiving Burden and Distinct Depressive-Symptom Trajectories From End-of-Life Caregiving Through the First 2 Bereavement Years

Potential Predictor	Minimal-Impact Resilience (*n*=133) *vs.*																	
	Recovery (*n* = 238)			Preloss-Only (*n* = 137)			Delayed Symptomatic (*n* = 46)			Relief (*n =* 37)			Prolonged Symptomatic (*n =* 41)			Persistently D.istressed (*n* = 17)		
	a*OR*	95% CI	*p*	a*OR*	95% CI	*p*	a*OR*	95% CI	*p*	a*OR*	95% CI	*p*	a*OR*	95% CI	*p*	a*OR*	95% CI	*p*
Trajectories of preloss caregiving burden																		
Slight	0.227	[0.024, 2.117]	.193	**0.023**	**[0.003, 0.203]**	**.001**	**0.058**	**[0.004, 0.762]**	**.030**	**0.003**	**[0.000, 0.037]**	**<.001**	**0.003**	**[0.000, 0.045]**	**<.001**	—	—	—
Mild	0.519	[0.057, 4.723]	.561	**0.082**	**[0.010, 0.658]**	**.019**	0.205	[0.018, 2.343]	.202	**0.009**	**[0.001, 0.078]**	**<.001**	**0.026**	**[0.003, 0.215]**	**.001**	**0.013**	**[0.001, 0.132]**	**<.001**
Moderate	0.750	[0.076, 7.444]	.806	0.482	[0.056, 4.160]	.507	0.318	[0.024, 4.202]	.384	**0.055**	**[0.006, 0.496]**	**.010**	0.131	[0.014, 1.205]	.073	0.127	[0.012, 1.330]	.085
Severe	Ref			Ref			Ref			Ref			Ref			Ref		
Preloss SOC + trajectories of preloss caregiving burden																		
SOC	**0.982**	**[0.965**, **1.000]**	**.048**	**0.940**	**[0.921, 0.959**]	**<.001**	**0.966**	**[0.942, 0.992]**	**.009**	**0.904**	**[0.877, 0.931]**	**<.001**	**0.940**	**[0.916, 0.966]**	**<.001**	**0.909**	**[0.874, 0.945]**	**<.001**
Trajectories of preloss caregiving burden																		
Slight	0.341	[0.035, 3.305]	.353	**0.100**	**[0.011, 0.931]**	**.043**	0.128	[0.009, 1.803]	.128	**0.030**	**[0.002, 0.402]**	**.008**	**0.011**	**[0.001, 0.204]**	**.003**	—	—	—
Mild	0.668	[0.072, 6.174]	.722	0.206	[0.025, 1.724]	.145	0.335	[0.028, 3.956]	.385	**0.042**	**[0.005, 0.381]**	**.005**	**0.064**	**[0.007, 0.562]**	**.013**	**0.054**	**[0.005, 0.607]**	**.018**
Moderate	0.827	[0.083, 8.241]	.871	0.692	[0.078, 6.163]	.742	0.384	[0.029, 5.131]	.469	**0.102**	**[0.011, 0.982]**	**.048**	0.188	[0.020, 1.772]	.144	0.229	[0.021, 2.547]	.230
Severe	Ref			Ref			Ref			Ref			Ref			Ref		

*Note.* Sample sizes in the slight, mild, moderate, and severe caregiving burden trajectories were 100, 370, 133, and 46, respectively. Bold indicates a significant association; Italics indicate no mediation effect; “—” indicates no a*OR* or 95% CI could be estimated due to small sample size. a*OR* = adjusted odds ratio; CI = confidence interval; Ref = references; SOC= sense of coherence.

**Table 3:** Mediation Effects of Preloss SOC on Associations Between End-of-Life Caregiving Burden Appraisal and Distinct Depressive-Symptom Trajectories From End-of-Life Caregiving Through the Initial Two Bereavement Years

Potential Predictor	Recovery (*n* = 238) vs.														
	Preloss-Only (*n* = 137)			Delayed Symptomatic (*n* = 46)			Relief (*n* = 37)			Prolonged Symptomatic (*n* = 41)			Persistently Distressed (*n =* 17)		
	a*OR*	95% CI	*p*	a*OR*	95% CI	*p*	a*OR*	95% CI	*p*	a*OR*	95% CI	*p*	a*OR*	95% CI	*p*
Trajectories of preloss caregiving burden															
Slight	**0.103**	**[0.027, 0.396]**	**.001**	0.256	[0.037, 1.777]	.168	**0.014**	***[0.002, 0.083]***	**<.001**	**0.011**	***[0.001, 0.115]***	**<.001**	—	—	—
Mild	**0.158**	**[0.048, 0.522]**	**.002**	0.395	[0.069, 2.249]	.295	**0.018**	***[0.005, 0.064]***	**<.001**	**0.049**	***[0.014, 0.177]***	**<.001**	**0.025**	***[0.005, 0.121]***	**<.001**
Moderate	0.642	[0.186, 2.216]	.484	0.424	[0.065, 2.789]	.372	**0.073**	***[0.019, 0.274]***	**<.001**	**0.175**	***[0.046, 0.670]***	**.011**	**0.170**	***[0.036, 0.797]***	**.025**
Severe	Ref			Ref			Ref			Ref			Ref		
Preloss SOC + trajectories of preloss caregiving burden															
SOC	**0.957**	**[0.942, 0.972]**	**<.001**	0.984	[0.962, 1.007]	.166	**0.920**	***[0.896, 0.946]***	**<.001**	**0.958**	***[0.935, 0.981]***	**<.001**	**0.925**	***[0.892, 0.960]***	**<.001**
Trajectories of preloss caregiving burden															
Slight	0.292	[0.071, 1.209]	.090	0.375	[0.050, 2.806]	.340	**0.088**	***[0.013, 0.608]***	**.014**	**0.032**	***[0.003, 0.342]***	**.004**	—	—	—
Mild	0.309	[0.090, 1.064]	.063	0.501	[0.085, 2.954]	.445	**0.063**	***[0.016, 0.245]***	**<.001**	**0.095**	***[0.025, 0.361]***	**.001**	**0.080**	***[0.015, 0.440]***	**.004**
Moderate	0.837	[0.236, 2.973]	.783	0.465	[0.070, 3.076]	.427	**0.123**	***[0.030, 0.497]***	**.003**	**0.227**	***[0.058, 0.891]***	**.034**	0.277	[0.055, 1.392]	.119
Severe	Ref			Ref			Ref			Ref			Ref		

*Note.* Sample sizes in the slight, mild, moderate, and severe caregiving burden trajectories were 100, 370, 133, and 46, respectively. Bold indicates significant association; Italics indicate no mediation effect; “---” indicates no a*OR* or 95% CI could be estimated due to small sample size. a*OR* = adjusted odds ratios; CI = confidence interval; Ref = references; SOC = sense of coherence.

**Table 4:** Mediation Effects of Preloss SOC on Associations Between Self-Perceived End-of-Life Caregiving Burden and Distinct Depressive-Symptom Trajectories From End-of-Life Caregiving Through the Initial Two Bereavement Years

Potential Predictor	Preloss-Only (*n* = 137) *vs.*												Prolonged Symptomatic (*n* = 41) *vs.*		
	Delayed Symptomatic (*n* = 46)			Relief *(n* = 37)			Prolonged Symptomatic (*n* = 41)			Persistently Distressed (*n* = 17)			Persistently Distressed (*n* = 17)		
	a*OR*	95% CI	*p*	a*OR*	95% CI	*p*	a*OR*	95% CI	*p*	a*OR*	95% CI	*p*	a*OR*	95% CI	*p*
Trajectories of preloss caregiving burden															
Slight	2.500	[0.389, 16.049]	.334	**0.133**	**[0.024**, **0.742]**	**.021**	0.111	[0.012, 1.048]	.055	—	—	—	—	—	—
Mild	2.500	[0.517, 12.093]	.255	**0.115**	**[0.041**, **0.319]**	**<.001**	**0.312**	**[0.110**, **0.885]**	**.029**	**0.156**	**[0.038**, **0.638]**	**.010**	0.500	[0.114, 2.186]	.357
Moderate	0.660	[0.119, 3.653]	.634	**0.113**	**[0.039**, **0.329]**	**<.001**	**0.273**	**[0.092**, **0.807]**	**.019**	**0.264**	**[0.070**, **1.001]**	**.050**	0.969	[0.232, 4.042]	.966
Severe	Ref			Ref			Ref			Ref			Ref		
Preloss SOC + trajectories of preloss caregiving burden															
SOC	**1.028**	**[1.004**, **1.053]**	**.021**	**0.962**	**[0.937**, **0.988]**	**.004**	1.001	[0.978, 1.025]	.942	0.967	[0.933, 1.003]	.069	0.966	[0.929, 1.005]	.090
Trajectories of preloss caregiving burden															
Slight	1.285	[0.183, 9.036]	.801	0.303	[0.050, 1.847]	.195	0.109	[0.011, 1.098]	.060	—	—	—	—	—	—
Mild	1.622	[0.320, 8.221]	.559	**0.205**	**[0.069**, **0.614]**	**.005**	***0.308***	**[*0.102***, ***0.931*]**	***.037***	*0.260*	[*0.058*, *1.169*]	*.079*	0.843	[0.171, 4.154]	.834
Moderate	0.555	[0.099, 3.114]	.504	**0.147**	**[0.049**, **0.438]**	**.001**	***0.271***	**[*0.090***, ***0.811*]**	***.020***	*0.331*	[*0.085*, *1.291*]	*.111*	1.221	[0.283, 5.274]	.789
Severe	Ref			Ref			Ref			Ref			Ref		

*Note.* Sample sizes in the slight, mild, moderate, and severe caregiving burden trajectories were 100, 370, 133, and 46, respectively. Bold indicates significant association; Italics indicate no mediation effect; “—” indicates no a*OR* or 95% CI could be estimated due to small sample size. AOR= adjusted odds ratios; CI= confidence interval; Ref = references; SOC= sense of coherence.

**Table 5:** Mediation Effects of Preloss SOC on Associations Between Self-Perceived End-of-Life Caregiving Burden and Distinct Depressive-Symptom Trajectories From End-of-Life Caregiving Through the Initial Two Bereavement Years

Potential Predictor	Delayed Symptomatic (*n*=46) vs.									Relief (*n*=37) *vs.*					
	Relief (*n* = 37)			Prolonged Symptomatic (*n* = 41)			Persistently Distressed (*n* = 17)			Prolonged Symptomatic (*n* = 41)			Persistently Distressed (*n* = 17)		
	a*OR*	95% CI	*p*	a*OR*	95% CI	*p*	a*OR*	95% CI	*p*	a*OR*	95% CI	*p*	a*OR*	95% CI	*p*
Trajectories of preloss caregiving burden															
Slight	**0.053**	**[0.006, 0.484]**	**.009**	**0.044**	**[0.003**, **0.621]**	**.021**	—	—	—	0.833	[0.066, 10.553]	.888	—	—	—
Mild	**0.046**	**[0.009, 0.233]**	**<.001**	**0.125**	**[0.024**, **0.643]**	**.013**	**0.063**	**[0.009**, **0.414]**	**.004**	2.727	[0.893, 8.326]	.078	1.364	[0.316, 5.892]	.678
Moderate	0.171	[0.029, 1.012]	.052	0.413	[0.069, 2.463]	.332	0.400	[0.057, 2.800]	.356	2.407	[0.736, 7.877]	.146	2.333	[0.567, 9.598]	.240
Severe	Ref			Ref			Ref			Ref			Ref		
Preloss SOC + trajectories of preloss caregiving burden															
SOC	**0.935**	**[0.906, 0.966]**	**<.001**	0.973	[0.945, 1.002]	.072	**0.940**	**[0.903, 0.979]**	**.003**	**1.040**	**[1.008**, **1.073]**	**.013**	1.005	[0.965, 1.047]	.801
Trajectories of preloss caregiving burden															
Slight	0.236	[0.023, 2.447]	.226	*0.085*	[*0.006*, *1.296*]	*.076*	—	—	—	0.359	[0.026, 5.031]	.447	—	—	—
Mild	**0.127**	**[0.023, 0.698]**	**.018**	*0.190*	[*0.035*, *1.040]*	*.056*	0.160	[0.022, 1.177]	.072	1.502	[0.444, 5.077]	.513	1.266	[0.262, 6.112]	.769
Moderate	0.265	[0.043, 1.624]	.151	0.488	[0.081, 2.956]	.435	0.596	[0.082, 4.350]	.610	1.845	[0.546, 6.229]	.324	2.253	[0.534, 9.512]	.269
Severe	Ref			Ref			Ref			Ref			Ref		

*Note.* Sample sizes in the slight, mild, moderate, and severe caregiving burden trajectories were 100, 370, 133, and 46, respectively. Bold indicates significant association; Italics indicate no mediation effect; “—” indicates no a*OR* or 95% CI could be estimated due to small sample size. a*OR* = adjusted odds ratios; CI= confidence interval; Ref = references; SOC = sense of coherence.

### Mediation Effects of Preloss SOC on Associations Between CRA Trajectories and Depressive-Symptom Trajectories Through the First 2 Years of Bereavement

When both SOC and CRA trajectories were included in the third step of the multinomial logistic regressions, stronger SOC was found to be associated with less distressing depressive-symptom trajectories in 15 of 21 comparisons (see the middle panels in Tables 2–5). Also, a stronger SOC was shown to be consistently associated with a greater likelihood of belonging to the minimal-impact resilience and recovery trajectories than the more distressing depressive-symptom trajectories (Tables 2 and 3). In addition, those participants with stronger SOC were identified as less likely to follow the relief trajectory (marked by moderate-to-severe symptoms during end-of-life caregiving) than the preloss-only, delayed-symptomatic, and prolonged-symptomatic trajectories, which involve substantially fewer symptoms during caregiving (Tables 4 and 5).

Including SOC in the multinomial logistic regressions substantially attenuated the associations between CRA and depressive-symptom trajectories, reducing the number of significant associations from 31 to 19 (see the bottom panels in Tables 2–5). For example, the greater likelihood of participants in the severe (vs. the slight and/or mild) CRA trajectory being in the preloss-only and delayed-symptomatic trajectories rather than the minimal-impact-resilience and recovery trajectories was almost completely attenuated (Tables 2 and 3). Also, the lower likelihood of participants in the slight (vs. severe) CRA trajectory being in the relief trajectory rather than the preloss-only (Table 4) and delayed-symptomatic (Table 5) trajectories was nullified. Furthermore, those participants in the moderate and mild CRA trajectories were no less likely than those in the severe CRA trajectory to be in the chronically persistent-distressed than in the recovery (Table 3) and delayed-symptomatic (Table 5) trajectories.

However, the effects of SOC were not significant on those participants in the mild and moderate *vs.* the severe CRA trajectories in terms of likelihood of being in the prolonged-symptomatic and chronically persistent-distressed trajectories rather than in the preloss-only trajectory (main effects and italics in Table 4). Nor were the effects of SOC significant on those in the slight and mild versus the severe CRA trajectories in terms of likelihood of being in the prolonged-symptomatic rather than the delayed-symptomatic trajectories (main effects and italics in Table 5). Therefore, in this study, SOC was not found to mediate these associations. For the remaining significant associations between CRA trajectories and depressive-symptom trajectories, SOC significantly attenuated association strength, as indicated by predominantly increased a*OR* and *p* values (Tables 2–5).

## Discussion

Over one in four (27.6%) of the participants in this study endured moderate-to-severe high end-of-life caregiving burdens, with those in lower-burden CRA trajectories associated with significantly higher SOC levels (Table 1), which is consistent with the findings of a 35-study systematic review (Del-Pino-Casado et al., 2019). The results of this study also support the cumulative-stress or wear-and-tear model of caregiving effects on bereavement adjustment (Schulz et al., 1997). Those in the severe CRA trajectory not only experienced higher subjective burden levels during the caregiving phase but also appeared emotionally depleted after the patient’s death. This depletion likely contributed to their heightened vulnerability to perceiving the loss as highly stressful, thereby increasing their likelihood of following more distressing depressive-symptom trajectories (i.e., relief, prolonged-symptomatic, and chronically persistent-distressed) than more-adaptive trajectories such as minimal-impact resilience, recovery, preloss-only, and delayed-symptomatic. These findings underscore the enduring psychological toll of severe subjective caregiving burden that extends into bereavement, elevating the risk of adverse mental health outcomes. Therefore, the results of this study support an urgent need for evidence-based interventions (Alam et al., 2020) that strengthen caregivers’ knowledge, skills, and self-efficacy in end-of-life care provision with the goals of reducing perceived burden, fostering emotional preparedness, and, ultimately, mitigating the risk of devolution into more severe depressive trajectories during bereavement.

Participants with stronger SOC were significantly more likely to follow minimal-impact resilience and recovery trajectories rather than more distressing trajectories, which is consistent with the findings of a previous meta-analysis (Del-Pino-Casado et al., 2019). Stronger SOC may enhance sense of comprehensibility, manageability, and meaningfulness throughout the caregiving and bereavement processes, thereby fostering psychological resilience and supporting recovery from the emotional burden of EOL caregiving and the grief associated with the loss of a loved one.

Furthermore, SOC was identified as significantly mediating the impact of caregiving burden on psychological distress during the first two bereavement years, fully or partially mediating most associations between CRA and depressive-symptom trajectories. As an inner coping resource, stronger SOC may allow caregivers to better manage stressful end-of-life caregiving by appraising it as more comprehensible, meaningful, and manageable (Antonovsky, 1993) and thus avoiding a wear-and-tear experience (Schulz et al., 1997). Therefore, SOC may be seen as a protective psychological factor that mediates the stress inherent in end-of-life caregiving to facilitate adjustment from end-of-life caregiving to bereavement. This protective/mediating effect of SOC merits notice because it completely eliminates or partially ameliorates the elevated likelihood of being in the four most distressing trajectories (preloss-only, relief, prolonged-symptomatic, and chronically persistent-distressed trajectories) rather than the milder depressive-symptom trajectories. This SOC-mediating effect was most notable among the participants in the most burdened CRA trajectory. This result is particularly important in light of current high health care costs and resource limitations (World Health Organization, 2020), suggesting the particular value of investing in interventions to boost SOC in those caregivers in the most burdened CRA trajectory. Health care professionals may help these caregivers mobilize resources and support through their social networks, nongovernmental patient-support organizations, and psychosocial professionals to help manage and accept the challenges of end-of-life caregiving. These interventions may reduce caregiver risk for the most distressing depressive-symptom trajectories during the first 2 years of bereavement, lowering the personal and health care costs of managing depressive symptoms (König, et al., 2019) and easing the financial strain (Ornstein et al., 2019) on health care systems (World Health Organization, 2020).

### Study Limitations

A key strength of this study is the longitudinal demonstration of how SOC mediates psychological distress in caregivers due to the intertwined stressors of end-of-life caregiving and bereavement, and lowers the risk of reverse causation. Grounded in the salutogenic model, which posits SOC as a mediator between stress and health outcomes, this study combines trajectory-based methods with longitudinal mediation to capture not only the presence of distress but its progression over time. By focusing on modifiable psychosocial factors, conceptual and methodological innovations are proposed that advance the current scientific understanding of caregiver adaptation in the context of end-of-life care. However, several limitations of this study warrant mention. The convenience sample of family caregivers of advanced cancer patients used in this study was recruited from a single Taiwanese hospital, which may limit generalizability, particularly in light of the significant influence that culture has on grief response. Moreover, the findings may not apply to caregivers bereaving patients with other diseases, dealing with sudden or traumatic deaths, or who are not the spouse or adult child of the care-recipient. Also, depressive symptoms were assessed using the CES-D rather than formal psychiatric diagnostic tools. Although this may overestimate severity, CES-D results ensure that caregiver distress and support needs are not overlooked. In addition, depressive-symptom trajectories were explored only through the initial 2 years of bereavement. This study followed Antonovsky’s original proposition that SOC is a relatively stable dispositional orientation. However, recent evidence suggests SOC may be modifiable over time and responsive to interventions (Guo et al., 2024; Langeland et al., 2022; Mittelmark et al., 2022; Schäfer et al., 2019). This raises important questions about the temporal stability of SOC. Future longitudinal and interventional studies are warranted to examine changes in SOC over time and assess its potential as a modifiable target for improving caregiver outcomes. Furthermore, small sample sizes for certain combined CRA and depressive-symptom trajectories may have led to imprecise estimates of SOC mediation. Finally, stressors and contextual factors beyond caregiver gender and age were not be accounted for in the depressive-symptom trajectories included in the stress–appraisal–coping model (Wen et al., 2021). As with most observational studies, unmeasured variables such as social support and coping strategies may influence outcomes. For instance, those caregivers with stronger SOC may have better leveraged their social networks to improve perceived social support, reducing their risk of distressing symptom trajectories.

### Clinical Implications

One in four caregivers of advanced cancer patients endured moderate-to-severely high EOL-caregiving burden and one-quarter (24.0%) experienced distressing depressive-symptom trajectories from EOL caregiving during the first two bereavement years. Boosting the SOC of these caregivers may reduce or even eliminate their relatively higher likelihood of being in the most distressing preloss and postloss depressive-symptom trajectories (preloss-only, delayed-symptomatic, relief, prolonged-symptomatic, and chronically persistent-distressed) rather than in milder depressive-symptom trajectories. Related high-quality end-of-life care interventions for caregivers hold the potential to not only alleviate the risk of profound, prolonged, and persistent depressive symptoms from end-of-life caregiving through bereavement but also benefit families and society (Blanner Kristiansen et al., 2019; Große et al., 2018; World Health Organization, 2020).

### Conclusions

Based on the findings of this study, SOC either fully or partially mediates the psychological impact of perceived end-of-life caregiving burden during the first 2 years of bereavement. SOC represents both a critical and actionable target in strategies designed to improve psychological well-being in caregivers caring for patients with cancer. Interventions that strengthen SOC may be key to enhancing the quality of EOL care and providing sustained support to caregivers in terms of alleviating their end-of-life caregiving burden and promoting psychological well-being across both the caregiving and bereavement periods.
